# Interesting Case of Uterine Hæmorrhage, Commencing before, and Continuing during Labour, to an Extent to Destroy the Child, as Well as Seriously to Jeopardize the Life of the Mother

**Published:** 1840-07-11

**Authors:** J. P. Curran

**Affiliations:** A Member of the Obstetric Class in the Philadelphia Dispensary


*Interesting Case of Uterine Hæmorrhage, commencing before, and continuing during Labour, to an extent to destroy the child, as well as seriously to jeopardize the life of the mother.* Reported by J. P. Curran, a Member of the Obstetric Class in the Philadelphia Dispensary.

Susan D——, æt. twenty-nine, in an advanced stage of her fifth pregnancy, was placed under my care for delivery, by my obstetric preceptor, Dr. Warrington.

At my introductory visit to her, I found her enjoying good health, and actively engaged in her household duties. On the 16th of April, 1840, however, I was called to her in consequence of considerable hæmorrhage, apparently from the uterus. I attempted to ascertain this by an examination per vaginam. Finding, on the introduction of the finger, the blood issuing copiously, I thought it best to apply a strong compress upon the vulva and perinæum. Visited her again two hours afterwards in company with Dr. Warrington; hæmorrhage had not increased. We introduced a tampon, gave her small doses of the solution of sulphate of morphia, and elevated the foot of the bed. In the evening she had considerable thirst. At an early visit in the morning of the 17th found her thirst increased, with foul breath, tongue dry, and covered with a white fur. In the evening she appeared to have less fever and hæmorrhage. I was called at 7 A. M., 18th, in consequence of the renewal of hæmorrhage, great faintness and thirst, and pains in her back and abdomen. She had removed the compress for the purpose of passing her urine, and had forced out the tampon by a severe cough. I re-applied these, and repeated the morphia. In the evening she was better, and had no renewal of the hæmorrhage. She remained comfortable during the greater part of the 19th, till 7 o'clock, P. M., when I was summoned in haste to see her. Found her in labour, and with considerable hæmorrhage. Dr. Warrington happened to call a few minutes after my arrival. On examination we were not able to arrive at a clear diagnosis whether it was a portion of the placenta, or a solid coagulum, which filled up the os tincæ. At this time she lost large quantities of blood whenever she moved, or had her position changed, however carefully. My friend and fellow member of Dr. W.'s obstetric class, C. C. Van Wyck, was now called in to participate in the interest and instruction which the case afforded. As the patient was considerably prostrated, and the uterine contractions feeble, though the os uteri appeared to be considerably dilated, at the suggestion of Dr. W. I gave her, in small and frequently repeated doses, a drachm or more of ergot suspended in brandy and cold water,—Dr. W. and Mr. Van Wyck making, at the same time, firm pressure upon the vulva and perinæum, to arrest the hæmorrhage, and prevent the too early rupture of the membranes.

The uterine contractions increased very slowly, and it was not until nearly four hours afterwards that it was thought proper to fix the patient for rupturing the membranes, or piercing the placenta, and terminating the labour by manual or instrumental aid. The hæmorrhage had been so profuse at every slight removal of the compression, that it was deemed unsafe to make frequent or extensive explorations till the parts of the patient were duly prepared. At this attempt, however, it was found that a portion of the placenta was stretched across the anterior part of the now fully dilated os uteri. The portion of the membranes situated over the posterior part of the orifice, was now ruptured, the hand passed into the cavity of the pelvis, and the child seized in the second position of the breech. During the few minutes the child occupied the os uteri, the hæmorrhage was completely arrested. The child was exsanguine, and dead, apparently from the profuse hæmorrhage, which had likewise prostrated the mother, rendering it necessary to administer brandy freely to support her. The placenta, which was delivered in eight or ten minutes afterward, had one-third of its disc covered, and its vessels congested with coagulated blood,—while the other portions appeared to have been subjected to the force of the contractions of the uterus, and recently separated from its surface.

The patient was now carefully bandaged, and placed up in bed; took solution of morphia, and several ounces of brandy, through the night. In the morning of the 20th, I found her with considerable after-pain, feeble pulse; continued treatment; in the evening she was more comfortable.

21*st.*—Gave her castor oil, with a few drops turpentine, she having had no alvine evacuations since the first occurrence of the hæmorrhage.

24*th.*—Had an attack of pain in the pubic region, which was relieved with hot brandy fomentations. She had reacted finely by the tenth day after delivery. She had very little lochia during her whole lying-in.

The attack of hæmorrhage was attributed by the nurse to her having imprudently lifted a heavy weight, which had so much contused the right mammary gland, that it was necessary to poultice it for several days.

